# The essential role of thoracic duct embolization in management of traumatic iatrogenic chylothorax

**DOI:** 10.1590/1677-5449.20230101

**Published:** 2023-10-30

**Authors:** Bruno Pagnin Schmid, Guilherme Moratti Gilberto, Marcela Juliano Silva Cunha, Leonardo Guedes Moreira Valle, Gustavo Foronda, Santiago Raul Arrieta, Felipe Nasser, Rodrigo Gobbo Garcia

**Affiliations:** 1 Hospital Israelita Albert Einstein - HIAE, São Paulo, SP, Brasil.; 2 Hospital Santa Marcelina, São Paulo, SP, Brasil.

**Keywords:** thoracic duct, lymphangiography, chylothorax, lymphatic diseases, interventional radiology, embolization, therapeutic, ducto torácico, linfografia, quilotórax, doenças linfáticas, radiologia intervencionista, embolização terapêutica

## Abstract

This study aims to describe a case series of patients who underwent thoracic duct embolization (TDE) to treat traumatic iatrogenic chylothorax (TIC). Three patients were included: Case #1, a 49-year-old woman with follicular lymphoma developed a TIC following video-assisted thoracoscopic surgery to resect a solid right paravertebral mass and was treated with TDE using microcoils and N-butyl cyanoacrylate (NBCA) glue. Case #2, a 68-year-old man with cardiac amyloidosis developed a TIC following heart transplantation and was treated with TDE using microcoils and ethylene vinyl alcohol copolymer. Case#3: A 6-year-old patient with congenital heart disease developed a TIC following a Fontan procedure and was treated with TDE using NBCA glue. All lesions were identified during lymphangiography and TDE was successfully performed in all cases. TDE is a safe and valuable technique that provides minimally invasive treatment for TCI.

## Resumo

Este estudo objetiva descrever uma série de casos de pacientes submetidos a embolização do ducto torácico (EDT) para tratamento de quilotórax iatrogênico (QI). Três pacientes foram incluídos. Caso 1: um homem de 49 anos com linfoma folicular apresentou QI após ressecção de uma massa paravertebral por toracoscopia vídeo-assistida e foi submetido a EDT com micromolas e n-butil-cianoacrilato (NBCA). Caso 2: um homem de 68 anos com amiloidose cardíaca apresentou QI após ser submetido a transplante cardíaco e foi submetido a EDT com micromolas e copolímero de etileno e álcool vinílico. Caso 3: um paciente de 6 anos com malformação cardíaca congênita apresentou QI após cirurgia de Fontan e foi submetido a EDT com NBCA. Todas as lesões foram identificadas durante a linfangiografia, e a EDT foi realizada com sucesso. A EDT é uma técnica segura e valiosa, que pode oferecer um tratamento minimamente invasivo em casos de QI.

## INTRODUCTION

Traumatic iatrogenic chylothorax (TIC) is a life-threatening condition associated with immunosuppression and malnutrition and can cause a high risk of mortality.^1,2^

It has a reported rate of 0.5% to 3% following thoracic surgery, with escalating incidence, due to the increased prevalence of more advanced thoracic resections as well as improved mortality among cancer patients.^2,3^

Treatment options consist of thoracic duct ligation, associated with high morbidity, or conservative management with dietary manipulation and octreotide, which usually involves a prolonged hospital-stay.^1^

Thoracic duct embolization (TDE) has emerged as a new therapy offering a minimally invasive approach to managing this debilitating condition with encouraging results, but it is still little known among medical professionals.^4^

This study aims to describe a case series of patients with TIC submitted to TDE, describing chylous leak imaging findings and technical aspects of the embolization procedures.

## CASE DESCRIPTION

This study was approved by the local institutional review board (Protocol number: 59697622.0.0000.0071. Consolidated opinion: 5.553.147). Informed consent was obtained.

This is a retrospective, single-center series of 3 consecutive cases of patients with TIC treated with TDE from November 2021 to August 2022 at a quaternary Hospital in Brazil. The patients’ characteristics and clinical presentations are shown in Table 1.

**Table 1:** Patients’ characteristics and clinical presentations.

**Case**	**1**	**2**	**3**
Operation Date	7 November 2021	27 December 2021	28 August 2022
Age (years)	49	68	6
Gender	Female	Male	Female
Primary disease	Follicular lymphoma	Cardiac amyloidosis	Congenital heart disease (*Double outlet right ventricle + inter-ventricular communication)*
Comorbidities	No	Multiple myeloma, smoking, DVT	DiGeorge syndrome
Surgery	Video-assisted thoracoscopic surgery to resect a solid right paravertebral mass	Heart transplantation	Fontan procedure
Chylous output before conservative management (mL/day)	300	800	340
Conservative treatment period (days)	7	12	9
Chylous output after conservative management (mL/day)	100	270	350
Clinical follow-up (months)	20.16	16.68	0.5

DVT: deep vein thrombosis.

Embolization was indicated after failed conservative management (chylous output >100mL/ day, for 7 days) with octreotide and dietary manipulation (a low-fat oral diet mainly consisting of fruits, vegetables, and whole grains).

All procedures were performed in the angiosuite of a quaternary care hospital using a fluoroscopic imaging unit (Philips Medical Systems®, Bothell, WA, United States) and were headed by 3 highly experienced interventional radiologists (more than 6 years of clinical practice) assisted by 2 interventional radiology fellows with the patient under general anesthesia in a supine position.

**Figure 1:**
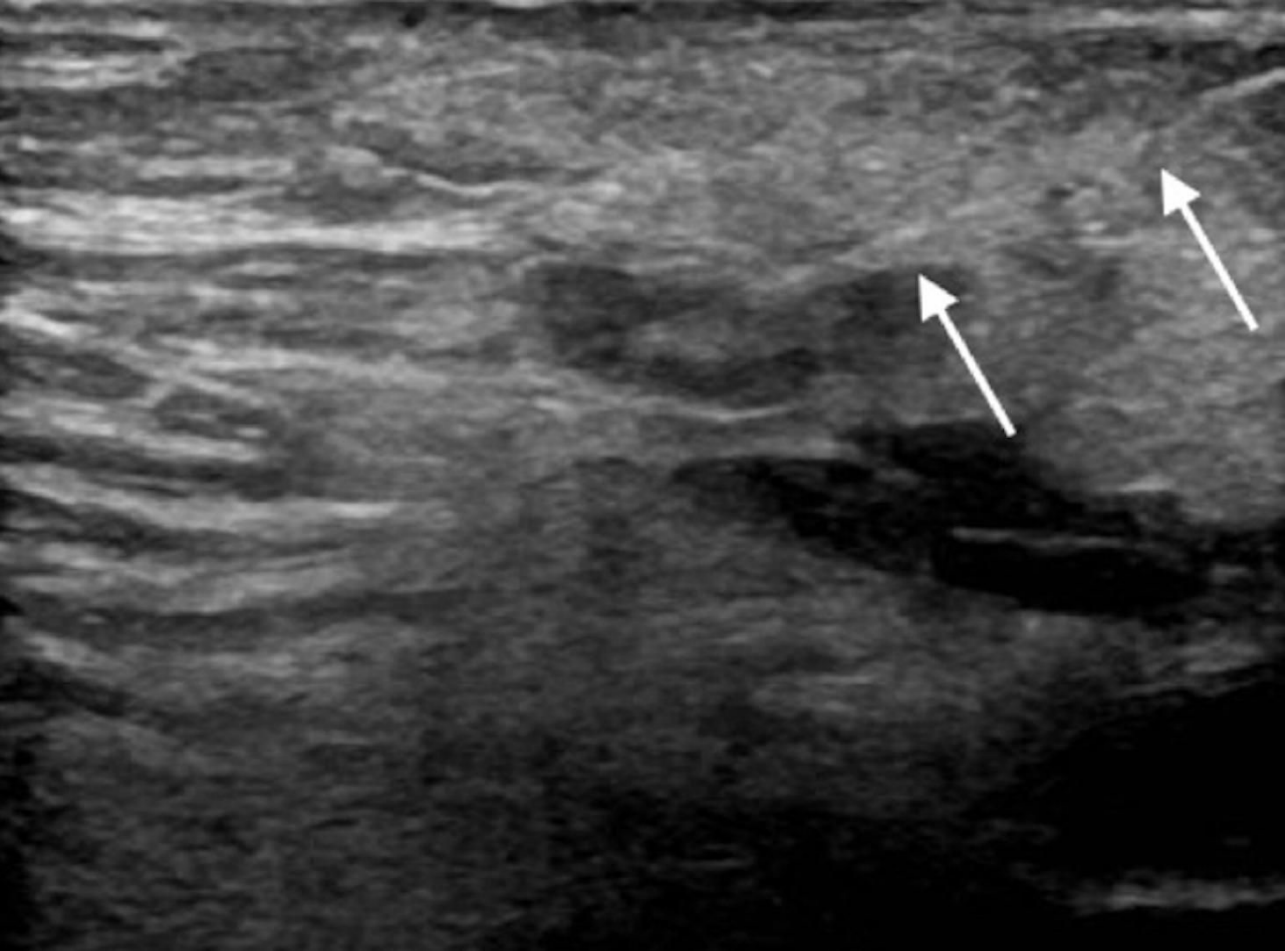
Groin lymph node puncture using a 25-gauge needle (white arrow) under ultrasound guidance.

Prophylactic cefazolin (1-2 g intravenously) was administered in all cases. Inguinal lymph nodes were accessed under ultrasound guidance with bilateral puncture using a 25-gauge needle. (Figure 1) Lipiodol infusion was performed with an angioplasty manometer, to control the injection pressure. A sequential compression device was applied on the lower limbs in order to speed up lymphatic drainage and provoke faster propagation of the contrast agent. A mean total contrast agent volume of 20mL was used in each case.

## CASE 1

A 49-year-old woman with follicular lymphoma developed a TIC following video-assisted thoracoscopic surgery to resect a solid right paravertebral mass. A lymphangiography was performed with injection of Lipiodol® (UltraFluide; Guerbet, Roissy, France) into the inguinal lymph nodes, enabling identification of the cisterna chyli.

Serial fluoroscopic imaging was used to monitor the progress of the ethiodized oil (Lipiodol®- UltraFluide; Guerbet, Roissy, France).

After visualization, the cisterna chyli was accessed by transhepatic percutaneous puncture under fluoroscopic and ultrasound guidance using a 22-gauge Chiba needle (Cook Medical, Bloomington, IN, United States). A Renegade Hi-Flo microcatheter (Stryker Neurovascular, Watertown, MA, United States) was advanced over a 0.018-inch V-18 ControlWire Steerable Guidewire (Boston Scientific, Marlborough, MA, United States) with successful catheterization into the thoracic duct.

**Figure 2:**
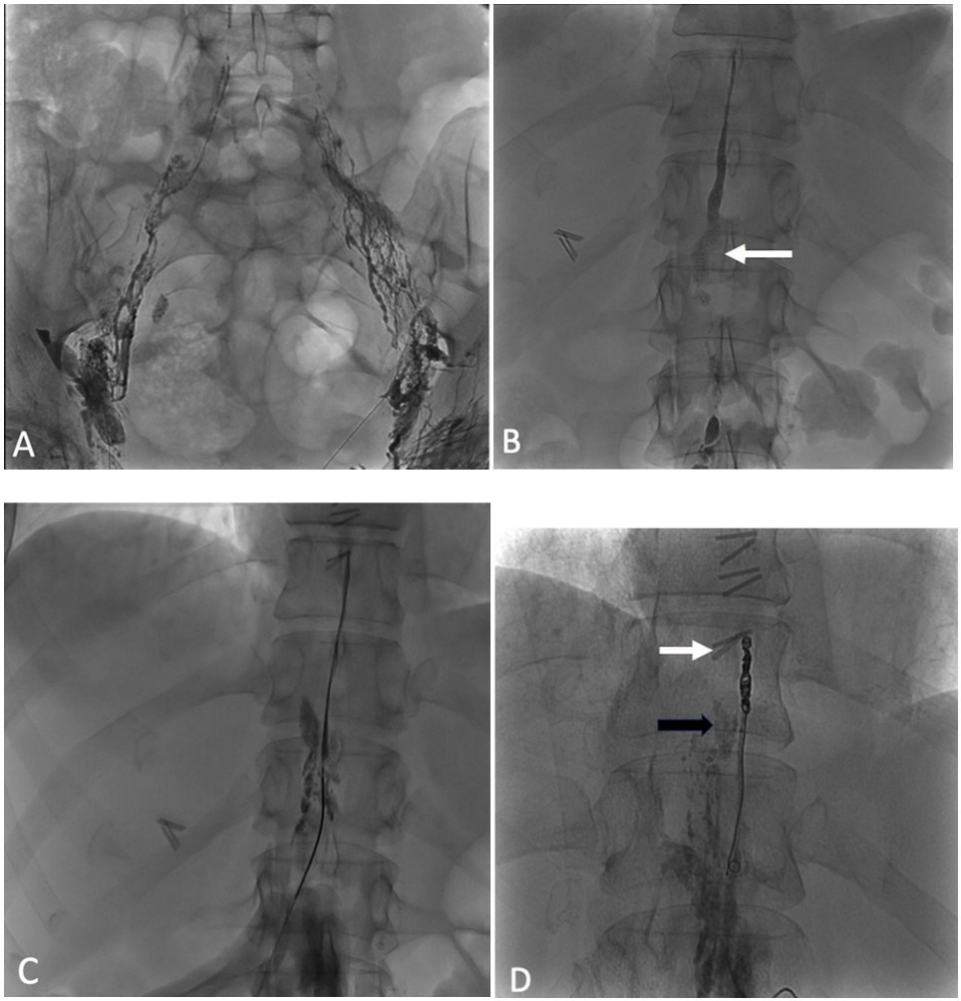
Thoracic duct embolization via percutaneous puncture of the cisterna chyli. (A) Serial fluoroscopic imaging showing the contrast agent ascending to abdominal lymph nodes; (B) Fluoroscopic imaging showing the cisterna chyli (white arrow); (C) Successful catheterization into the thoracic duct; (D) Fluoroscopic imaging following coil embolization (white arrow) and glue embolization (black arrow).

After fluoroscopic visualization of the leak, embolization was performed using microcoils. The microcatheter was flushed with 5% dextrose solution followed by an injection of N-butyl cyanoacrylate (TruFill; Codman and Shurtleff, Raynham, MA, United States) (NBCA) in a solution of ethiodized oil (Lipiodol, Guerbet, Villepinte, France) at a proportion of 1/2 (Figure 2).

The patient presented complete resolution of symptoms. Two days after the procedure, the chest tube was removed and she was discharged home. There was no recurrence during a 1.68-year follow-up.

## CASE 2

A 68-year-old man with cardiac amyloidosis developed a TIC following heart transplantation. No leaks were visualized and no distinct cisterna chyli was identified after lymphangiography using inguinal lymph nodes for access.

Thus, the thoracic duct was cannulated at its confluence with the left subclavian vein via a direct, cervical percutaneous access under ultrasound guidance using a 22-gauge needle.^5^ Correct needle positioning was confirmed by ultrasound visualization and fluoroscopy.

Next, a Renegade Hi-Flo microcatheter (Stryker Neurovascular, Watertown, MA, United States) was advanced over a 0.018-inch V-18 ControlWire Steerable Guidewire (Boston Scientific, Marlborough, MA, United States) to cannulate the thoracic duct.

**Figure 3:**
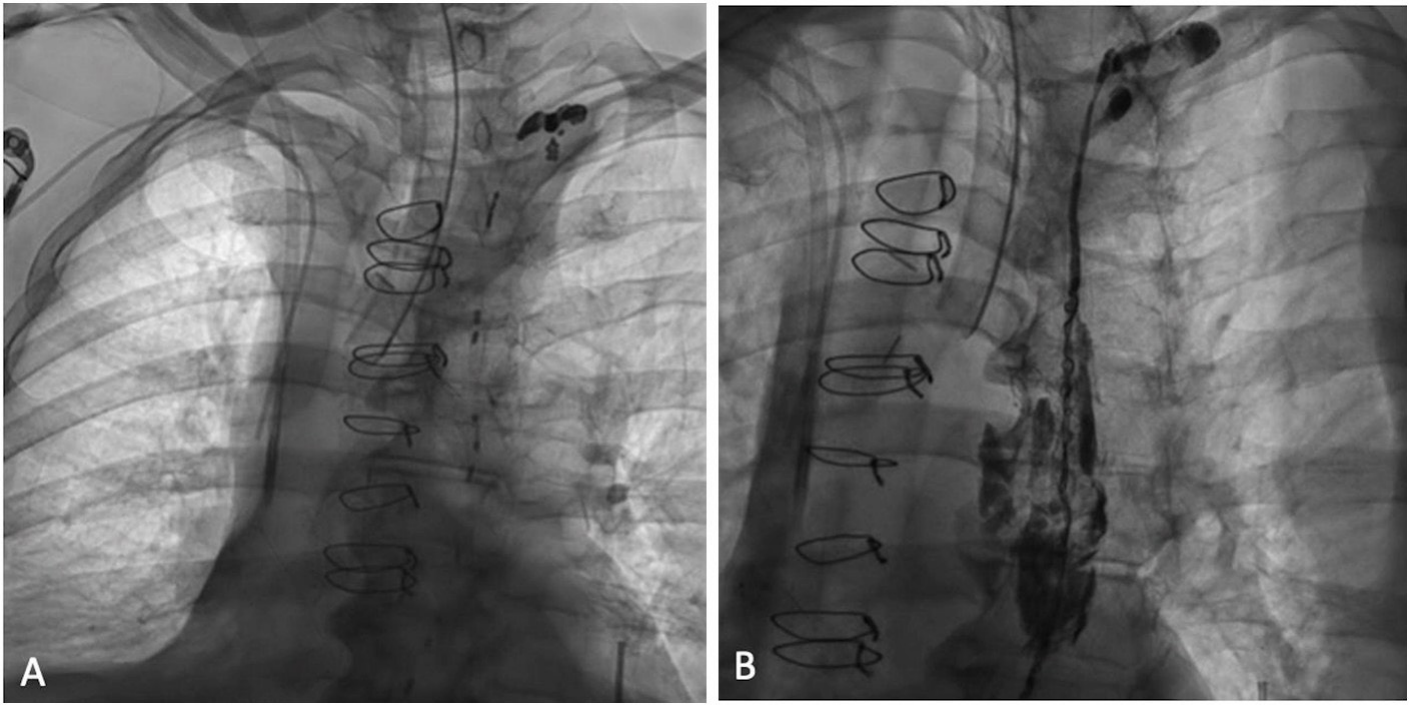
Thoracic duct embolization via percutaneous puncture of the thoracic duct. (A) Fluoroscopic imaging showing the thoracic duct cannulated at its confluence with the left subclavian vein via a direct percutaneous access; (B) Fluoroscopic imaging following coil and liquid agent embolization.

After confirmed retrograde cannulation, fluoroscopic imaging demonstrated visualization of the leak, and embolization was performed using microcoils and Onyx (a liquid embolization agent consisting of ethylene vinyl alcohol) (Medtronic, Minneapolis, MN, United States) (Figure 3).

The patient presented a decrease in the amount of chylous output after TDE (200mL/ day), but underwent thoracic duct ligation 15 days after the procedure due to re-accumulation of pleural fluid. There was no recurrence during a 1.39-year follow-up.

## CASE 3

A 6-year-old patient with congenital heart disease developed a TIC following a Fontan procedure. A chylous leak was identified, after lymphangiography using inguinal lymph nodes for access, confirming the thoracic duct injury.

The left brachial vein was punctured and cannulation of the thoracic duct was attempted using a 2.7-Fr microcatheter (Progreat^®^, Terumo, Shibuya-ku, Tokyo, Japan) passed through a 4-Fr Vertebral catheter (Cordis, Miami Lakes, FL, United States), without success.

**Figure 4:**
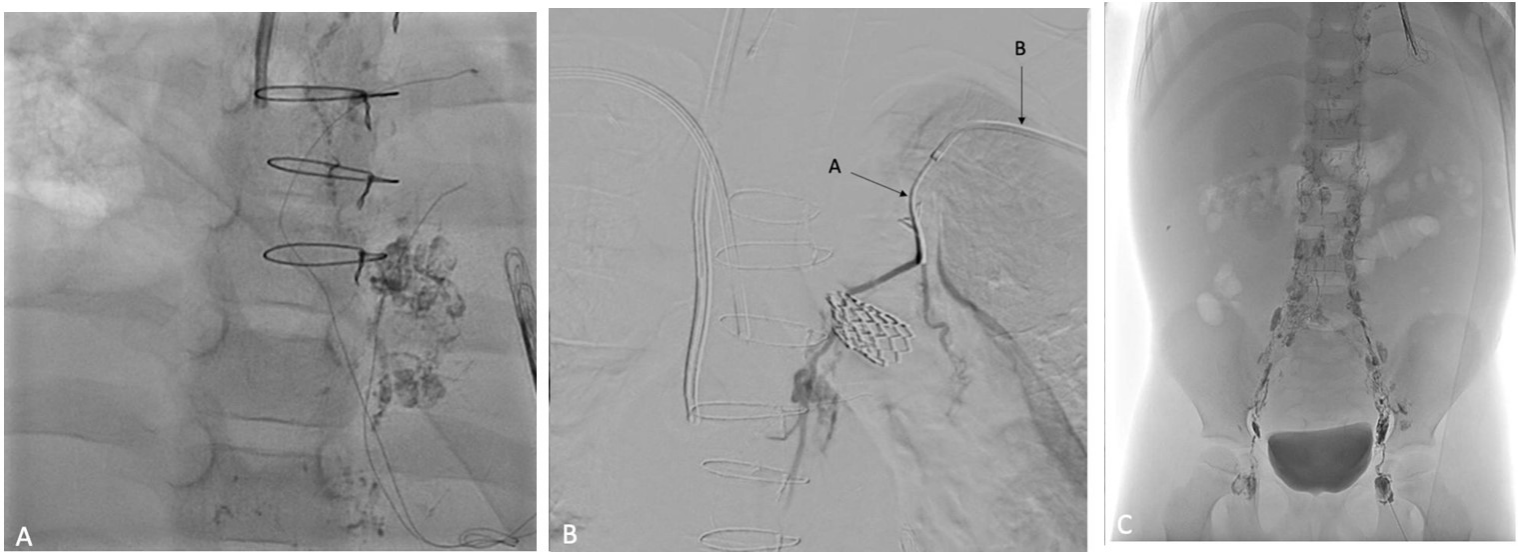
Thoracic duct embolization using the transvenous approach. (A) Fluoroscopic imaging showing the chylous leak, confirming the thoracic duct lesion; (B) The transvenous approach: Thoracic duct cannulation was attempted using a microcatheter (A) passed through a macrocatheter (B), after left brachial vein access; (C) Final lymphangiography demonstrating no chylous leak.

Embolization was therefore performed via the inguinal access using ethiodized oil (Lipiodol, Guerbet, Villepinte, France) to promote more distal embolization, enabling it to reach the leak site. Competition lymphography showed a significant reduction of the leak identified previously (Figure 4). The chest tube was removed 9 days after the procedure, and she was discharged home 15 days after TDE.

All lesions were identified during lymphangiography and TDE was successfully performed in all cases. No complications were reported during the postoperative period. Details of the embolization procedures are shown in Table 2.

**Table 2:** Details of embolization procedures.

**Case**	**1**	**2**	**3**
Access	Inguinal lymph nodes/cisterna chyli	Inguinal lymph nodes/direct, percutaneous, cervical thoracic duct	Inguinal lymph nodes/left brachial vein
Embolic agent	Interlock Microcoils (Boston Scientific, Marlborough, MA, United States) + NBCA	Interlock Microcoils (Boston Scientific, Marlborough, MA, United States) + Onyx (Medtronic, Minneapolis, MN, United States)	Ethiodized oil (Lipiodol, Guerbet, Villepinte, France)
Total operating time (hours)	2	3.5	3
Fluoroscopy time (minutes)	39.47	68.19	80.19
Cumulative Air Kerma (mGy)	820.59	1488.65	326.15

NBCA: N-butyl cyanoacrylate.

## DISCUSSION

This case series confirms TDE as a valuable technique that must be known to medical professionals involved in management of patients presenting chylothorax. It offers a minimally invasive approach to this challenging condition with promising results.

Conservative treatment with dietary manipulation is based on restricting long-chain fatty acid intake and replacing it with medium-chain triglycerides.^6^ Successful resolution of chylous fistula has been described with enteral nutrition and total parenteral nutrition based on this strategy.^7,8^

Octreotide (a long-acting somatostatin analogue) adds a complementary effect in this conservative management of chylothorax and some authors report a success rate of 87-90% using it as an adjunct to conservative treatment.^9^

Etilefrine is a sympathomimetic agent that causes contraction of thoracic duct smooth muscle and is under investigation as another medication for use in non-invasive management, but is yet to demonstrate improvements in clinical success.^10-12^

However, these approaches demand a long hospital-stay, as seen in all cases, in which the mean duration of conservative treatment was 9.3 days, with better results for low-output fistulas. Therefore, TDE emerges as a helpful technique to enable rapid hospital discharge and, presumably, reduce costs to health services and improve patient treatment experiences and general satisfaction.

Another treatment method is surgical exploration, including pleurodesis and thoracic duct ligation, with or without clipping of lymphatic sites.^13^ These techniques provide definitive lymphostasis with high technical success, reaffirming their crucial role, especially after failed TDE, and as was necessary in patient #2.^13^ On the other hand, they are also associated with potential morbidity and mortality, especially in debilitated patients.^3^

In such situations, TDE emerges as a solution with lower complication rates.^12^ In a recent systematic review, one group of authors reported a 4-6% rate of minor complications (further chyle leak, leg and pedal edema, asymptomatic pulmonary embolization, and inconsequential coil misplacement).^12^ In comparison, rates of minor complications following thoracic surgical ligation were higher: pneumonia (28.5-33%), wound infection or dehiscence (21.4%), and prolonged thoracostomy drainage (6.6%).^12^ No complications were observed in our series, reaffirming the potential benefit of this minimally invasive technique.

The first lymphatic intervention technique involving thoracic duct embolization was described by Cope et al.^14^ in the late 1990s. Since then, several techniques have been proposed to optimize results.^3,15-17^

The success of these procedures depends on recognition of important landmarks. The lymphatic system consists of 3 parts that drain different segments of the body: soft tissue lymphatic system, intestinal lymphatic system, and liver lymphatic system.^18^ They communicate with each other and usually join together at the level of the cisterna chyli and continue as the thoracic duct.^18^ Moreover, the main lympho-venous connection is between the thoracic duct and the junction of the left subclavian and jugular veins.^18^

The original pedal lymphangiography was replaced by inguinal intranodal lymphangiography, allowing a much shorter procedure.^3,15^ Use of sequential compression devices also speeds up the procedure. Both strategies were used in this series, with satisfactory results (mean operating time: 2.83hours).

Besides, novel retrograde transvenous and percutaneous cervical thoracic duct cannulation techniques have been instituted as bail-out methods. These options are more technically challenging, but extremely helpful in situations in which intranodal lymphangiography is not possible or fails to identify the cisterna chyli, improving the clinical success of TDE, as observed in patients #2 and #3.^19-22^

Regarding an accurate evaluation of the efficacy of lymphatic interventions for chylothorax, a metanalysis including 407 patients from 9 studies was conducted. The pooled clinical success rate of TDE was 79.4% (95% CI, 64.8%-89.0%; *I*^2^ = 68.1%).^23^

All the studies included in this metanalysis used coils and NBCA as embolic agents.^21^ There is no consensus regarding the ideal embolic agent in TDE, but this embolization protocol was also used in patient #1. First, coils were placed to provide a matrix for NBCA polymerization and to avoid non-target embolization. The liquid embolic agent Onyx (Medtronic, Minneapolis, MN, United States) was used in patient #2 due to its more predictable distribution, in order to mitigate the risk of inadvertent embolization, since direct percutaneous access was used and the puncture site was in close proximity to the left subclavian vein.

Finally, in cases in which cannulation of the thoracic duct is not feasible, ethiodized oil can be used alone, promoting a more distal distribution of the embolic agents and enabling a successful therapeutic procedure, as observed in case #3.

In conclusion, TDE is a safe and valuable technique for minimally invasive treatment of traumatic iatrogenic chylothorax.
